# Continuation of dual anti-platelet therapy after drug-eluting stents in primary angioplasty beyond 12 months

**DOI:** 10.12688/f1000research.1-53.v1

**Published:** 2012-11-21

**Authors:** Iman Mohasseb, Christian A Gericke

**Affiliations:** 1 Department of Cardiology, Plymouth Hospitals NHS Trust, Plymouth, PL6 8DH, UK; 2 PenCLAHRC, National Institute for Health Research, Peninsula Medical School, Plymouth, PL4 8AA, UK; 3 The Wesley Research Institute, Brisbane, QLD 4066, Australia

## Abstract

In this correspondence we discuss the results of the meta-analysis by De Luca *et al. *(2012) in the Archives of Internal Medicine which found that late myocardial reinfarction and stent thrombosis is more common in drug-eluting stents than in bare-metal stents. We discuss the clinical implications of this paper for dual anti-platelet therapy which did not receive sufficient attention in the original publication and the accompanying editorial.

## Correspondence

The meta-analysis by De Luca *et al.* ^1^ showed that the incidence of late (> 2 years) myocardial reinfarction and stent thrombosis is significantly higher in drug-eluting stents (DES) compared to bare-metal stents (BMS) in primary angioplasty despite the significant reduction in long-term target vessel revascularization associated with DES.

While the Comment section of the paper briefly mentions the role of more potent and prolonged dual anti-platelet therapy in countering these worrisome findings, the related Commentary ^2^ does not. However, in our view the current practice of discontinuing dual anti-platelet therapy after 12 months in DES in most patients is the most likely explanation for the observed increase in late stent thrombosis and reinfarction incidence, in concordance with pathological evidence that even beyond 40 months, DES do not fully epithelialize ^3^. In the De Luca *et al.* ^1^ meta-analysis, the DES survival curves for both reinfarction and stent thrombosis start diverging from the BMS curves one year after stent implantation until year 6.

This also raises the most relevant question for practitioners: should we prolong dual anti-platelet therapy beyond 12 months after DES implantation? The Dual Antiplatelet Therapy Study (DAPT) is expected to give us a definitive answer to this question in 2014 ^4^. For the time being, it seems that the argument to continue dual anti-platelet therapy beyond 12 months, which is fully in line with the current ACCF/AHA/SCAI recommendation ^5^ to continue dual anti-platelet therapy *for at least* 12 months after DES implantation, has gained in strength.

## References

[1] De Luca G Dirksen MT Spaulding C: Drug-Eluting vs Bare-Metal Stents in Primary Angioplasty: A Pooled Patient-Level Meta-analysis of Randomized Trials. *Arch Intern Med.* 2012;172(8):611–21 10.1001/archinternmed.2012.758 22529227

[2] Brophy JM: Drug-Eluting Stents in ST-Segment Elevation Myocardial Infarction: Comment on “Drug-Eluting vs Bare-Metal Stents in Primary Angioplasty”. *Arch Intern Med.* 2012;172(8):621–2 10.1001/archinternmed.2012.694

[3] Joner M Finn AV Farb A: Pathology of drug-eluting stents in humans: delayed healing and late thrombotic risk. *J Am Coll Cardiol.* 2006;48(1):193–202 10.1016/j.jacc.2006.03.042 16814667

[4] U.S. National Institutes of Health [cited 8 May 2012]. Reference Source

[5] Levine GN Bates ER Blankenship JC: 2011 ACCF/AHA/SCAI Guideline for Percutaneous Coronary Intervention. A report of the American College of Cardiology Foundation/American Heart Association Task Force on Practice Guidelines and the Society for Cardiovascular Angiography and Interventions. *J Am Coll Cardiol.* 2011;58(24):e44–122 10.1016/j.jacc.2011.08.007 22070834

