# Supplementary material for: Continuation of dual anti-platelet therapy after drug-eluting stents in primary angioplasty beyond 12 months
Source: F1000Res. 2012 Nov 21;1:53. [Version 1] doi: 10.12688/f1000research.1-53.v1 (PMC3799550; doi:10.12688/f1000research.1-53.v1)
Supplement: Peer review history

# Peer review: Referee response for version 1

**Published:** 2012-12-05

**Authors:** Bruce Biccard

**Affiliations:** 1 Department of Anaesthetics, Nelson R Mandela School of Medicine University of Kwazulu-Natal, Durban, South Africa

I have read this submission. I believe that I have an appropriate level of expertise to confirm that it is of an acceptable scientific standard.

# Peer review: Referee response for version 1

**Published:** 2012-11-29

**Authors:** Sripal Bangalore

**Affiliations:** 1 Department of Medicine, Cardiology Division, New York University School of Medicine, New York, NY, USA

I have read this submission. I believe that I have an appropriate level of expertise to confirm that it is of an acceptable scientific standard.
